# Nasopharyngeal brushing: a convenient and feasible sampling method for nucleic acid-based nasopharyngeal carcinoma research

**DOI:** 10.1186/s40880-018-0278-z

**Published:** 2018-04-12

**Authors:** Pei-Fen Zhang, Xiao-Hui Zheng, Xi-Zhao Li, Tian Tian, Shao-Dan Zhang, Ye-Zhu Hu, Wei-Hua Jia

**Affiliations:** 1 Tumor Resource Bank, State Key Laboratory of Oncology in South China, Collaborative Innovation Center for Cancer Medicine, Sun Yat-sen University Cancer Center, 651 Dongfeng East Road, Guangzhou, 510060 Guangdong P. R. China; 2 Affiliated Tumor Hospital of Xinjiang Medical University, Ürümqi, 830000 Xinjiang P. R. China

**Keywords:** Nasopharyngeal brushing, Tissue samples, Nasopharyngeal carcinoma, Nucleic acid, Cancer research

## Abstract

**Background:**

Tissue specimens for nasopharyngeal carcinoma (NPC) research are scarce because of sampling difficulties. Previous studies have suggested non-invasive nasopharyngeal brushing as an effective sampling method for NPC diagnosis. The present study aimed to evaluate the feasibility of nasopharyngeal brushing in the acquisition of NPC nucleic acids for research.

**Methods:**

Nasopharyngeal brushing samples were acquired from 24 healthy individuals and 48 NPC patients. Tissues from 48 NPC and 18 nasopharyngitis patients were collected by endoscopic biopsy. The expression levels of tumor suppressor genes (TSGs) and Epstein–Barr virus (EBV)-encoded microRNAs as well as EBV DNA copy number were measured by quantitative polymerase chain reaction in both types of samples.

**Results:**

Among six TSGs examined, the expression levels of two genes were significantly decreased in nasopharyngeal brushing and tissue samples from NPC patients as compared with those from healthy/nasopharyngitis individuals. Four EBV-encoded microRNAs, mir-bart1-5p, mir-bart5, mir-bart6-5p, and mir-bart17-5p, were significantly up-regulated in both NPC brushing and tissue samples compared with those from healthy/nasopharyngitis controls (*P *< 0.001). EBV DNA was significantly increased in both nasopharyngeal brushing samples (*P *< 0.001) and tissue samples (*P *< 0.001) from NPC patients in comparison with those from healthy controls.

**Conclusions:**

Nasopharyngeal brushing can obtain sufficient tumoral materials for the analysis of viral nucleic acid, including EBV-encoded microRNAs and EBV DNA. For the detection of TSG expression, nasopharyngeal brushings was feasible but inferior to tissue samples. This study confirms nasopharyngeal brushing as an applicable sampling method that can aid in nucleic acid-based NPC research.

**Electronic supplementary material:**

The online version of this article (10.1186/s40880-018-0278-z) contains supplementary material, which is available to authorized users.

## Background

Nasopharyngeal carcinoma (NPC) is highly prevalent in South China, where 20–50 per 100,000 individuals are at risk of developing NPC [1–4]. According to the World Health Organization criteria, NPC is classified into three histological types, among which undifferentiated carcinoma is the most prevalent type in South China and strongly related to Epstein–Barr virus (EBV) infection [5, 6]. The strong etiological link between EBV infection and NPC can be distinctly reflected by the abnormal viral DNA load and deregulation of EBV gene expression in tumor cells [7–10]. The multiple-repeat EBV genomic sequence, BamHI-W, has been extensively used for NPC detection with a high sensitivity [6, 8]. EBV-encoded BamHI-A rightward transcript (BART) microRNAs have been found to be highly expressed in NPC patients and closely related to NPC progression [7, 9]. Previous study has also reported EBV-encoded mRNAs combined with EBV DNA load as useful biomarker for NPC diagnosis [11]. In addition, the development of NPC can also be attributed to epigenetic factors. Promoter hypermethylation of properly validated tumor suppressor genes (TSGs) has been increasingly implicated as an early indicator in NPC carcinogenesis [12, 13]. Therefore, the investigation of virus- and tumor-associated nucleic acids for NPC screening and diagnosis has become a hot spot in cancer research.

Despite the high incidence of NPC, tissue resources for NPC research are not enough. Unlike many other carcinomas, NPC lesions are quite small and rarely require surgery. Currently, the most reliable tissue-sampling method is endoscopic biopsy on suspected tumor sites. However, small biopsy samples are usually applied for histopathological examination on a priority basis and rarely left for scientific purposes after diagnosis [14, 15]. Besides, tissue samples from healthy individuals are not available because of the invasiveness. Body fluids such as blood and urine do not directly contact neoplastic sites and therefore may not be specific enough for NPC detection [16, 17]. In most clinical situation, only small numbers of cancer cells are present in body fluids. Therefore, a more sensitive and specific method is required to fully support NPC research [18].

Nasopharyngeal brushing, known as a non-invasive and convenient tissue-sampling method, comes into direct contact with the neoplastic site and can obtain exfoliative epithelium or tumor cells as previously demonstrated [10, 19]. It has been applied to large-scale EBV screening and NPC diagnosis at the level of EBV-related nucleic acids, including DNA, mRNA, and microRNA, in high-risk populations [9, 13, 17, 19–22]. The specificity and sensitivity of EBV-related biomarkers in nasopharyngeal brushings are quite high [8, 9, 11, 22]. Alternative approaches to detecting tumor gene promoter hypermethylation in nasopharyngeal brushings have been verified for early NPC detection, with higher sensitivity and specificity than detecting EBV DNA markers in plasma [13, 21, 23, 24]. The use of brush cytology for NPC detection has also been evaluated, although the cytologic pickup is inferior to that obtained by biopsy [25, 26]. These observations indicate the strong possibility of nasopharyngeal brushing in acquiring sufficient tumor cells and viral nucleic acid for NPC analysis.

To establish the low-invasive nasopharyngeal brushing method for robust use in translational NPC research, we carried out a systematic investigation of tumor-associated genes and viral nucleic acid in both nasopharyngeal brushing samples and tissue samples. The expression of six well-known NPC TSGs and four EBV microRNAs as well as the copy number of EBV BamHI-W were evaluated in this study.

## Methods

### Collection of brushing samples

Nasopharyngeal brushing samples collected from 48 NPC patients and 24 healthy individuals at Sun Yat-sen University Cancer Center (SYSUCC) between April 19, 2013 and August 03, 2015 were used for TSG analysis. Another 20 pairs of nasopharyngeal brushing samples from NPC patients and healthy participants were collected during the same period for EBV DNA and microRNAs analysis. Under the guidance of endoscopy by experienced physicians, a nasopharyngeal brush (Copan Diagnostics, Murrieta, CA, USA) was inserted into the nasopharyngeal cavity and rotated several times over the nasopharyngeal epithelium at the site of the suspected focus. Immediately after sampling, the brush tip (1.5 cm) was cut and placed in 1 mL of RNAlater solution (Invitrogen, Carlsbad, CA, USA) and stored at − 80 °C until use. All brushing samples were stored in the Tumor Resource Bank of SYSUCC and approved by the Human Ethics Committee of SYSUCC. All participants provided informed consent.

### Collection of tissue samples

Nasopharyngeal tissue samples were collected from 66 participants suspected of NPC in the head and neck clinic at SYSUCC between January 05, 2009 and March 03, 2010. Among the participants, 18 were eventually diagnosed with chronic nasopharyngitis, and 48 exhibited biopsy-positive NPC. An endoscope was slowly stretched into the patient’s nasopharynx, and images were captured at the site of suspicious lesions. Tissues were collected and pretreated with 1 mL of RNAlater (Invitrogen), then drained and stored at − 80 °C. All samples were stored in the Tumor Resource Bank of SYSUCC. The Human Ethics Committee of SYSUCC approved this study. All participants provided informed consent.

### Brush cytology

Nasopharyngeal brushing samples were smeared onto pathological slides, fixed in 10% formalin, and stained with hematoxylin–eosin (HE) or subjected to in situ hybridization (ISH) for EBV-encoded small RNAs (EBERs). The smears were observed using an upright microscope (Nikon; NI-SS, 933394, Chiyoda-Ku, Tokyo, Japan).

### Histo-morphology experiments

Tissues were fixed in 10% formalin, embedded in paraffin, sliced into 3–4 μm slices, and either stained with HE or subjected to ISH for EBERs. Sections were observed using an upright microscope (Nikon).

### EBER 1/2 RNA hybridization

The presence of EBV in tumor cells was assessed by ISH using the EBER ISH kit (ZSGB Bio-tech Co Ltd., Beijing, China). ISH was performed according to the manufacturer’s instructions.

### RNA extraction and quantitative polymerase chain reaction (qPCR) analysis of RNA expression

Total RNA was extracted from nasopharyngeal brushing and tissue samples using TRIzol reagent (Invitrogen) following the manufacturer’s instructions. For microRNA inspection, a total of 350 ng RNA from each nasopharyngeal brushing sample or 100 ng from each tissue sample was used for reverse transcription with a TaqMan (MicroRNA) Reverse Transcription Kit (Applied Biosystems, Foster City, CA, USA) according to the manufacturer’s protocol. Reverse transcription reactions (20 μL volume/sample) were conducted with custom stem-loop primers (Applied Biosystems) specific to the corresponding mature sequence obtained from miRBase (http://www.miRBase.org, Additional file 1: Table S1). U6 small nuclear RNA (RNU6B) was used as an endogenous control. Four EBV microRNAs, mir-bart1-5p, mir-bart5, mir-bart6-5p, and mir-bart17-5p, were used as targets. Relative expression levels of the target microRNAs were calculated after normalizing to RNU6B. All primers were synthesized by Life Technologies, Inc. (Gaithersburg, MD, USA).

To examine the expression of 6 TSGs, positive regulatory domain I-binding factor 1 and retinoblastoma protein-interacting zinc-finger gene domain-containing protein 5 (*PRDM5*), calcium channel regulatory subunit α2δ3 (*CACNA2D3*), retinoblastoma protein-interacting zinc finger gene 1 (*RIZ1*), checkpoint with forkhead and ring finger domains (*CHFR*), WNT inhibitory factor 1 (*WIF1*), and Ras association domain family 1A (*RASFF1A*), 500 ng of total RNA was reversely transcribed into cDNA and amplified using SYBR Green Master Mix (Bio-Rad, Hercules, CA, USA) following the manufacturer’s protocol. Primer design was performed with Primer BLAST. The primer sequences are summarized in Table 1. qPCR was performed on a CFX96 Real-Time PCR System (Bio-Rad). Relative expression levels of the TSGs were calculated after normalization to the reference gene, ribosomal protein S13 (RPS13).

**Table 1:** Primer and probe sequences used in qPCR for TSGs and EBV BamHI-W detection

Primer	Sequences	Tm (°C)	Amplicon length (bp)
CACNA2D3	Forward, 5′-CAGCTGCCTCTGTGAATCTGTGG-3′	63.58	288
	Reverse, 5′-ATGATTTAGCATGCCAAGAGAACATC-3′	60.24	
PRDM5	Forward, 5′-CAGGTTCTCCCTGAAGTCCT-3′	58.35	249
	Reverse, 5′-TGAGATGGTGCCTCATGAAC-3′	57.59	
WIF1	Forward, 5′-TGAATTCCTGTCCTTGCGCT-3′	59.96	296
	Reverse, 5′-ACTCGCAGATGCGTCTTTCA-3′	60.04	
RIZ1	Forward, 5′-TGAATCAGAACACTACTGAGCC-3′	57.81	297
	Reverse, 5′-GCAGCCAGTTTCCCTTCTCT-3′	59.96	
CHFR	Forward, 5′-AACCGGAACACAGGTCTGG-3′	59.55	247
	Reverse, 5′-TGGACGGTTTGGGCATTTCT-3′	60.18	
RASFF1A	Forward, 5′-CGCGCATTGCAAGTTCACC-3′	61.08	242
	Reverse, 5′-AGCCTGTGTAAGAACCGTCC-3′	59.68	
RPS13	Forward, 5′-TCGGCTTTACCCTATCGACGCAG-3′	64.47	153
	Reverse, 5′-ACGTACTTGTGCAACACCATGTGA-3′	63.24	
BamHI-W	Forward, 5′-CCCAACACTCCACCACACC-3′	60.53	76
	Reverse, 5′-TCTTAGGAGCTGTCCGAGGG-3′	60.40	
Hybridization probe	5′-FAMCACACACTACACACACCCACCCGTCTC-TAMRA-3′		

*qPCR* quantitative polymerase chain reaction, *EBV* Epstein–Barr virus, *CACNA2D3* calcium channel regulatory subunit α2δ3, *PRDM5* positive regulatory domain I-binding factor 1 and retinoblastoma protein-interacting zinc-finger gene domain-containing protein 5, *WIF1* WNT inhibitory factor 1, *RIZ1* retinoblastoma protein-interacting zinc finger gene 1, *CHFR* checkpoint with forkhead and ring finger domains, *RASFF1A* Ras association domain family 1A, *RPS13* ribosomal protein S13

### DNA extraction and qPCR analysis of EBV DNA copy number

Total DNA from nasopharyngeal brushing and tissue samples was extracted using an automated workstation (Chemagic Star; Hamilton Robotic, Bonaduz, GR, Switzerland) following the manufacturer’s protocol. DNA concentration was quantified using a NanoDrop 1000 Spectrophotometer (NanoDrop Technologies, Waltham, MA, USA). qPCR was performed based on a previously mature fluorogenic PCR system [27]. In this system, amplification primers targeting the BamHI-W region of EBV DNA genome and a dual-labeled hybridization probe were included. The sequences are shown in Table 1. The standard ladders, which contained the BamHI-W region of the EBV genome (10^2^,10^3^, 10^4^, 10^5^, 10^6^, and 10^7^ copies/2 μL) were used to obtain the standard curve. Each PCR was set up in a reaction volume of 8 μL, including 4 μL PCR master mix, 1 μL primers, 0.2 μL probe, 0.8 μL water, and 2 μL DNA template. Thermal cycling was initiated with a denaturation step of 5 min at 95 °C, and then 45 cycles of 95 °C for 15 s, 60 °C for 30 s, and 72 °C for 15 s were carried out. The EBV DNA levels in brushing and tissue samples are expressed as copies/ng DNA.

### Statistical analysis

The 2 − ∆Ct method was used to calculate the expression of target genes relative to suitable reference genes. Statistical significance was determined using the unpaired two-tailed Mann–Whitney U test or Student’s t tests. P < 0.05 was considered statistically significant. The fold change of expression level was calculated using the formula 2 − (∆CT[case] −  ∆CT[control]). Statistical analyses were performed using GraphPadPrism5.0 (GraphPad software, Inc., La Jolla, CA, USA) and Excel software (Microsoft Corporation, Redmond, Washington D.C., USA).

## Results

### Histological and cytological observations of tissue sections and brushing smears

**Fig. 1:**
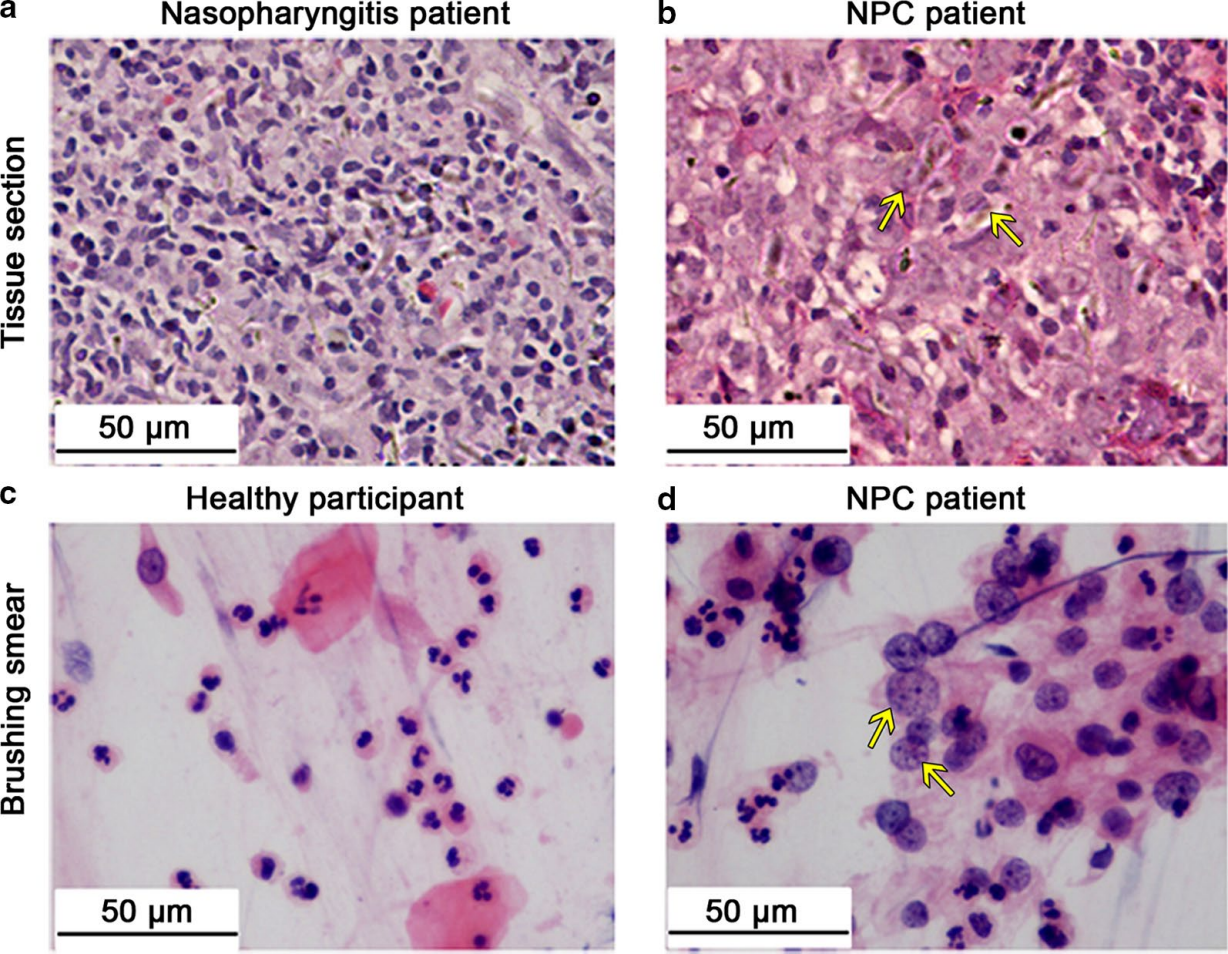
Hematoxylin–eosin (HE) staining of nasopharyngeal tissue sections and brushing smears (40×). **a** Nasopharyngeal tissue from a nasopharyngitis patient shows inflammatory nasopharyngeal epithelium infiltrated with plenty of lymphocytes. **b** Poorly differentiated nasopharyngeal carcinoma (NPC) tissue shows tumor nests surrounded with lymphocytes. **c** Brushing smear sample from a healthy participant shows normal nasopharyngeal mucosa, consisting of columnar cells, squamous cells, and lymphoid cells. **d** Brushing smear sample from a NPC patient shows typical NPC cells with large nuclei, a high nuclear/cytoplasmic ratio, and prominent nucleoli. Arrows indicate tumor cells

Tissue sections from nasopharyngitis patients showed inflammatory epithelium infiltrated with large amounts of lymphocytes (Fig. 1a), whereas nasopharyngeal tissue sections from NPC patients showed obvious tumor nests, surrounded by infiltrating lymphocytes (Fig. 1b). In brushing smears, normal nasopharyngeal mucosa from healthy participants presented typical columnar cells, squamous cells, and lymphoid cells (Fig. 1c), whereas in nasopharyngeal mucosa from NPC patients, a considerable number of exfoliative tumor cells mixed with a few normal epithelial cells and lymphocytes were observed (Fig. 1d). These results indicated that nasopharyngeal brushing could indeed harvest cytological constituents for examination and confirmed that the tissue and brushing samples collected were qualified for further study.

### Expression levels of TSGs in nasopharyngeal brushing and tissue samples

**Fig. 2:**
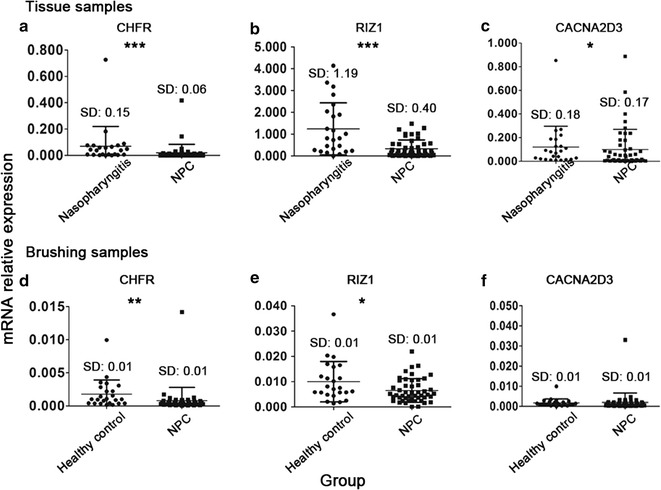
Expression levels of tumor suppressor genes calcium channel regulatory subunit α2δ3 (CACNA2D3), retinoblastoma protein-interacting zinc finger gene 1 (RIZ1), and checkpoint with forkhead and ring finger domains (CHFR) in nasopharyngeal brushing and tissue samples. The relative expression levels of CACNA2D3 (**a**, **d**), CHFR (**b**, **e**), and RIZ1 (**c**, **f**) in nasopharyngeal brushing and tissue samples from healthy controls, nasopharyngitis patients, and NPC patients were measured by real-time quantitative polymerase chain reaction. Data were analyzed by the Mann–Whitney *U* test. *SD* standard deviation. *P < 0.05, **P < 0.01, ***P < 0.001

Among the six TSGs examined, the relative expression levels of CHFR, RIZ1, and CACNA2D3 in tissue samples were significantly decreased in NPC patients compared with those in nasopharyngitis patients (all *P *< 0.05; Fig. 2a–c). However, there was no significant difference in the expression levels of PRDM5, WIF1, and RASFF1A between these two groups (data not shown). In nasopharyngeal brushing samples from 48 NPC patients (Table 2), the expression levels of CHFR and RIZ1 were significantly down-regulated as compared with those in samples from healthy controls (both *P *< 0.05; Fig. 2d, e). No significant difference was observed in the expression of CACNA2D3 (Fig. 2f), PRDM5, WIF1, or RASFF1A (data not shown). The fold changes of CHFR, RIZ1, and CACNA2D3 expression were 0.18, 0.21, and 0.40 in tissue samples and 0.42, 0.63, and 0.69 in brushing samples, respectively. These results suggested that nasopharyngeal brushings was applicable but inferior to tissue samples for TSGs detection.

**Table 2:** Tumor stage of NPC patients who underwent nasopharyngeal brushing

Variable	Number of patients
Total	48
Clinical stage	
I/II	3
III	17
IV	11
T stage	
1	1
2	3
3	18
4	9
N stage	
0	2
1	10
2	14
3	5
M stage	
0	30
1	1

The pathological staging information was evaluated by clinical doctors based on comprehensive results of magnetic resonance imaging (MRI), histopathologic examination, and clinical symptoms. Some patients were biopsy-diagnosed with NPC in our cancer center but subsequently moved to other hospitals for further diagnosis and treatment. For these patients, results such as MRI were not collected, while only their histopathological information was obtained. The staging information of 17 patients was lost in this study

*NPC* nasopharyngeal carcinoma

### ISH detection of EBERs in tissue sections and brushing smears

**Fig. 3:**
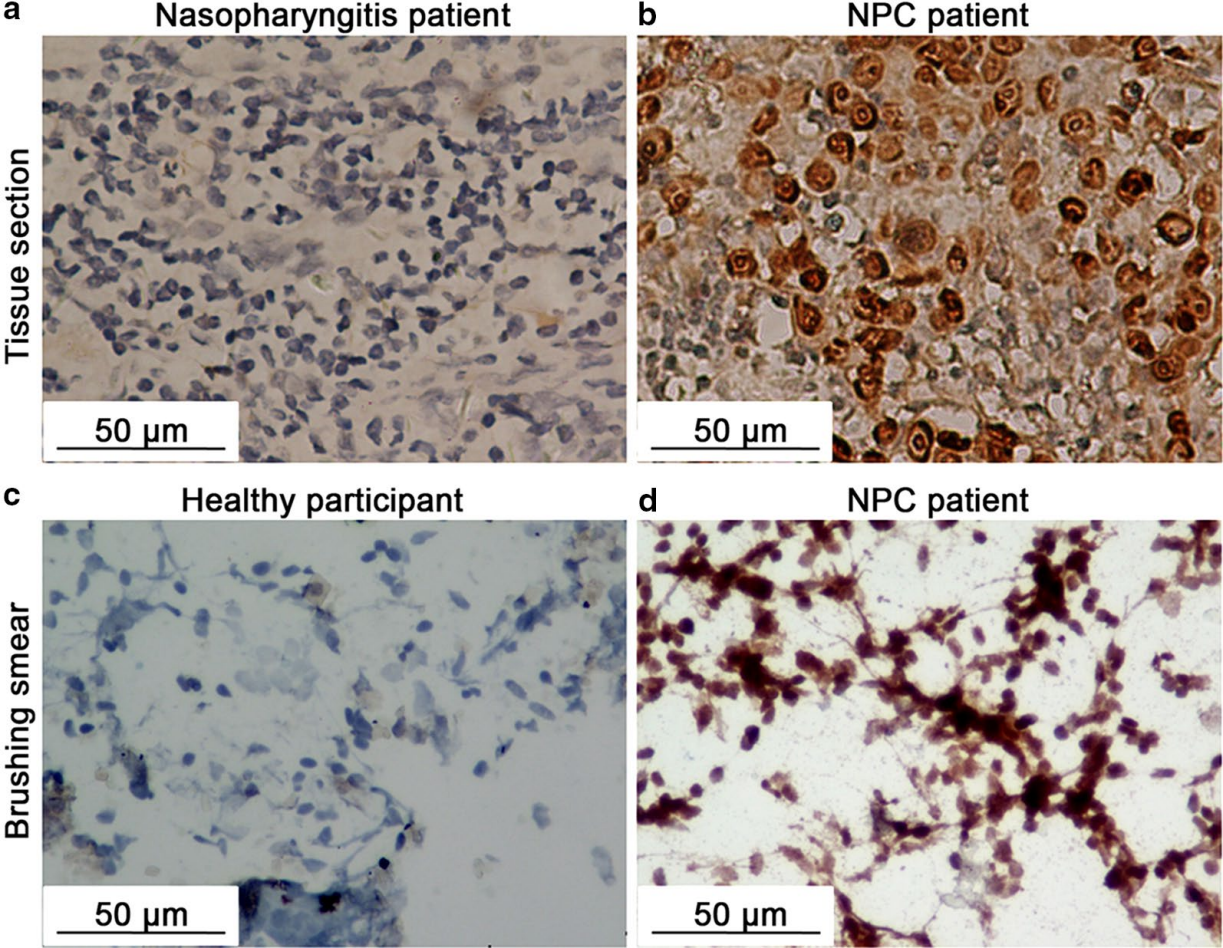
In situ hybridization of EBV-encoded small RNAs (EBERs) in nasopharyngeal tissue sections and brushing smears (40×). **a** Nasopharyngeal tissue from a nasopharyngitis patient shows no EBERs expression. **b** Nasopharyngeal tissue slice from a NPC patient shows strong signal of nuclear localization of EBERs within tumor cells. **c** Brushing smear sample from a healthy participant shows no EBERs signal. **d** Brushing smear sample from a NPC patient shows high EBERs expression in tumor cells

In order to confirm the presence of EBV in brushing and tissue samples, we carried out ISH detection of EBER 1/2. Tissue sections from nasopharyngitis patients showed little EBER 1/2 signal (Fig. 3a), while in tissue slices from NPC patients, strong EBER 1/2 signals localizing within tumor nuclear were observed (Fig. 3b). Similarly, nasopharyngeal brushing smears from healthy individuals showed no EBER signal as expected (Fig. 3c). However, in brushing smears from NPC patients, high signal of nuclear localization of EBER 1/2 within tumor cells was found (Fig. 3d). These results verified the existence of EBV both in nasopharyngeal tissue and brushing samples, and confirmed that the samples were qualified for subsequent EBV-related nucleic acid study.

### Expression levels of EBV-encoded microRNAs in nasopharyngeal brushing and tissue samples

**Fig. 4:**
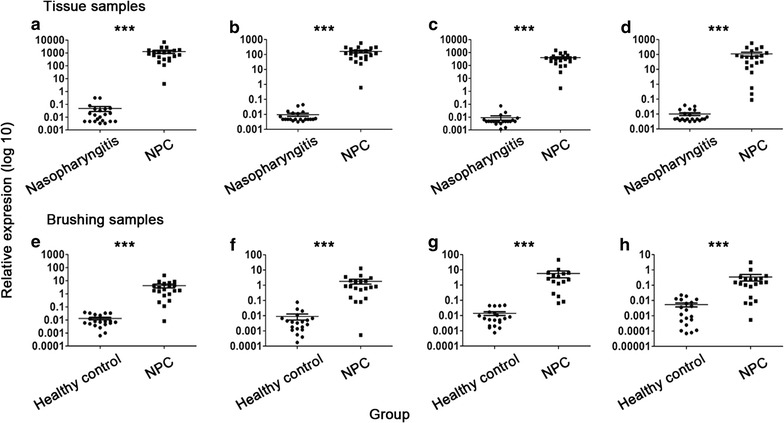
Expression levels of four Epstein–Barr virus (EBV)-encoded microRNAs in nasopharyngeal brushing and tissue samples. The expression levels of mir-bart1-5p (**a**), mir-bart5 (**b**), mir-bart6-5p (**c**), and mir-bart17-5p (**d**) in nasopharyngeal tissue samples from nasopharyngitis and NPC patients as well as the expression levels of mir-bart1-5p (**e**), mir-bart5 (**f**), mir-bart6-5p (**g**), and mir-bart17-5p (**h**) in nasopharyngeal brushing samples from healthy controls and NPC patients were measured by qPCR. Data were analyzed by Student’s *t* test. *P < 0.05, **P < 0.01, ***P < 0.001

The expression levels of mir-bart1-5p, mir-bart5, mir-bart6-5p, and mir-bart17-5p were significantly up-regulated in NPC tissues compared with those in nasopharyngitis controls (all *P *< 0.001; Fig. 4a–d), among which mir-bart1-5p was the most obviously up-regulated. In nasopharyngeal brushing samples, the expression levels of all four microRNAs were also significantly up-regulated in NPC patients compared with those in healthy controls (all *P *< 0.001; Fig. 4e–h). These results indicated that EBV-encoded microRNAs could be obtained and detected in nasopharyngeal brushing samples.

### EBV BamHI-W copy number in nasopharyngeal brushing and tissue samples

**Fig. 5:**
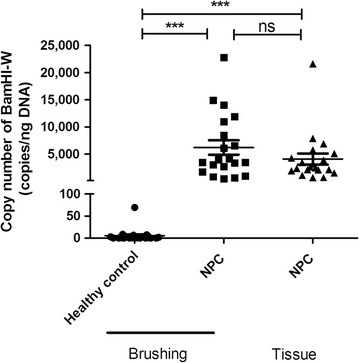
Copy number of EBV-encoded BamHI-W in nasopharyngeal brushing and tissue samples. The copy number (copies/ng DNA) of EBV-encoded BamHI-W in nasopharyngeal brushing and tissue samples from healthy controls and NPC patients was measured by qPCR. Data were analyzed by Student’s *t* test. ***P < 0.001. *ns* no significance

To examine the feasibility of using nasopharyngeal brushing samples to detect EBV DNA, we tested the copy number of EBV BamHI-W in both tissue and brushing samples. As shown in Fig. 5, BamHI-W was barely detectable in brushing samples from healthy controls, with a mean of 5.1 copies/ng DNA, except for a 43-year-old male healthy participant with positive serum viral capsid antibody (VCA-IgA). His brushing sample contained 69.7 copies/ng DNA. In brushing samples, BamHI-W was detectable in all NPC patients, with a mean copy number of 6221.1 copies/ng DNA, which was significantly higher than that in healthy controls (*P *< 0.001). Tissues from NPC patients yielded a mean copy number of 4087.0 copies/ng DNA, which was significantly higher than that in brushing samples from healthy controls (*P* < 0.001). Although the mean copy number of BamHI-W in tissue samples was lower than that in brushing samples from NPC patients, the difference was not significant. These results confirmed that components of EBV DNA in nasopharyngeal brushing samples were consistent with those in tissue samples and that nasopharyngeal brushing may serve as a promising non-invasive sampling method in EBV DNA-related NPC determination.

## Discussion

In the present study, EBV-encoded microRNAs (mir-bart1-5p, mir-bart5, mir-bart6-5p, and mir-bart17-5p) and EBV BamHI-W were easily detectable in both nasopharyngeal brushing and tissue samples from NPC patients. For the detection of CHFR, RIZ1, and CACNA2D3, brushing samples were applicable but inferior to tissue samples. The present study provides strong evidence that non-invasive nasopharyngeal brushing can be applied to support nucleic acid-based NPC research.

With the advantages of convenience in handling, non-invasiveness, easy acceptance by patients, low cost, and repeatability over nasendoscopic tissue biopsy, nasopharyngeal brushing has attracted increasing attention for more than a decade. Plenty of studies have revealed that nasopharyngeal brushing samples are available for EBV DNA, microRNAs, and mRNA detection in patients suspected of NPC [8, 9, 11, 21, 28]. In accordance with these observations, EBER ISH results in the present study showed high EBER signaling in both brushing smears and tissue sections from NPC patients, while no signaling from healthy and nasopharyngitis controls was observed (Fig. 3). Additionally, we observed significant increases of EBV microRNA levels and EBV DNA copy number in brushing samples from NPC patients (Figs. 4, 5).

The abundance of microRNAs in nasopharyngeal brushing samples was lower than that in nasopharyngeal tissue samples, which may be due to insufficient sampling. As shown in Fig. 1, brushing smears contain less tumor cells than tissue sections. These results are supported by previous brushing cytology studies [25, 26]. An anti-viral capsid antigen (VCA)-IgA-positive healthy person showed no aberrant EBV-encoded microRNA levels in brushing samples (data not shown), suggesting EBV-encoded microRNAs as specific biomarkers for NPC detection.

We and others [11, 21, 28] also detected EBV DNA in nasopharyngeal brushing samples from healthy participants. This result verifies that EBV-infected B lymphocytes have a homing preference for the nasopharyngeal region (Waldeyer’s ring) and that the virus is shed into the oropharyngeal space [29, 30]. Conversely, some previous studies showed a complete absence of EBV DNA in nasopharyngeal brushing samples donated by healthy EBV carriers [20, 21, 31], which is likely due to inadequate sampling of the nasopharynx or insensitive qPCR assays in healthy controls.

Studies have reported that TSG hypermethylation can be detected in nasopharyngeal brushing samples and suggested the potential role of nasopharyngeal brushing in the early diagnosis of NPC [13, 21, 23, 32]. Different from qualitative methylation-specific PCR carried out in those studies, we performed qPCR to verify the differential expression of TSGs. Among the six selected TSGs, CHFR, RIZ1, and CACNA2D3 were significantly suppressed in NPC patients, suggesting that qPCR can be applied to auxiliary screening for potential TSGs. For the other three TSGs, no significance was observed between NPC and healthy/nasopharyngitis controls, which may be due to different methylation status, since earlier studies have reported that the promoter methylation frequency of individual TSGs is variable in NPC patients [12, 13].

Trends of TSG expression in brushing samples were in accordance with those in nasopharyngeal tissues. Two highly suppressed TSGs in tissue samples, CHFR and RIZ1, were significantly suppressed in nasopharyngeal brushing samples obtained from NPC patients, whereas CACNA2D3, which was significantly down-regulated in tissues, did not differ significantly between NPC patients and healthy controls in nasopharyngeal brushing samples. This may be due to the low abundance of tumor cells in nasopharyngeal brushing samples as reported previously [26] and evidenced by our cytology results (Fig. 1d), indicating that the abundance of non-NPC cells can affect TSG detection in brushing samples. In summary, nasopharyngeal brushing is inferior to tissue biopsy for qPCR detection of TSGs, but can still be applied to auxiliary screening for potential TSGs.

Although nasopharyngeal brushing is becoming increasingly popular for NPC diagnosis, it has limitations, such as inadequate sampling, anatomical nasal obstruction, missing small tumors, presence of septal deviation, turbinate hypertrophy preventing brush insertion, pollution, and low specificity [26]. We and others have reported low tumor cell acquisition rates, even under the guidance of a nasopharyngeal endoscope [25, 26]. Nevertheless, a convenient sampling method should not require endoscopic visualization of the nasopharynx for accurate placement. Modifications to trans-nasal brushes, such as changing the existing shape to a bulbous configuration and attaching hairs at the tip of the plastic knob, may improve the efficiency of current nasopharyngeal brushing. Moreover, to prevent nasal contamination and increase sample purity, brush with a sliding plastic sheath has been designed [26]. A previously described trans-oral method of nasopharyngeal brushing achieved high sensitivity and specificity for the detection of NPC [33]. This method is more comfortable and non-invasive than trans-nasal brushing and can overcome the disadvantages of nasal obstruction and contamination. However, the efficiency of trans-oral brushing seems to depend largely on the guidance of the nasopharyngeal screen in the study. Moreover, trans-oral nasopharyngeal sampling requires customized brushes with angled heads, which are more difficult to produce and need further modification. Besides, it seems difficult to access the nasopharynx and to apply sufficient brushing pressure by trans-oral method. Further training of experienced brushers is required.

We should acknowledge a few potential drawbacks of our study. First, most patients recruited were at stage III–IV. It is not yet clear whether nasopharyngeal brushing samples from early-stage patients can be applied to nucleic acid-based research. Previous studies have reported that EBV-encoded microRNA levels and EBV DNA loads from nasopharyngeal brushing samples are related to tumor stage [8, 9, 34]. It will be of great significance to investigate the feasibility of nasopharyngeal brushing in early-stage patients for NPC detection. Moreover, due to objective reasons, the sample size used in our study was small, and brushing samples were taken only once from the nasal cavity of NPC patients or healthy individuals. To fully illustrate the application of nasopharyngeal brushing to cancer research, further studies with more abundant and repeated brushing samples should be performed.

## Conclusions

Nasopharyngeal brushing can obtain sufficient tumoral materials for the analysis of viral nucleic acid, including EBV-encoded microRNAs and EBV DNA. For the detection of TSG expression, nasopharyngeal brushing was feasible but inferior to tissue biopsy and needs further investigation. This study confirms the use of nasopharyngeal brushing as a feasible sampling method in NPC-related nucleic acid research.

## Additional file

**Additional file 1: Table S1.** Sequences of Epstein–Barr virus-encoded microRNAs.
