# Supplementary material for: Nasopharyngeal brushing: a convenient and feasible sampling method for nucleic acid-based nasopharyngeal carcinoma research
Source: Cancer Commun (Lond). 2018 Apr 12;38:8. doi: 10.1186/s40880-018-0278-z (PMC5993107; doi:10.1186/s40880-018-0278-z)
Supplement: Supplementary file 1 — Additional file 1: Table S1. Sequences of Epstein–Barr virus-encoded microRNAs. [file 40880_2018_278_MOESM1_ESM.docx]

**Table S1: Sequences of Epstein-Barr virus-encoded microRNAs**

| microRNA | Sequences |
| --- | --- |
| mir-bart1-5p | UCUUAGUGGAAGUGACGUGCUGUG |
| mir-bart-5 | CAAGGUGAAUAUAGCUGCCCAUCG |
| mir-bart6-5p | UAAGGUUGGUCCAAUCCAUAGG |
| mir-bart17-5p | UAAGAGGACGCAGGCAUACAAG |
